# Long Noncoding RNA AF131217.1 Regulated Coronary Slow Flow-Induced Inflammation Affecting Coronary Slow Flow via KLF4

**DOI:** 10.21470/1678-9741-2020-0573

**Published:** 2022

**Authors:** Haibing Jiang, Zhengrong Ge, Lijing Zhang, Yi Yang, Xueqin Zhai, Zhanxi Chen, Qing Wei

**Affiliations:** 1 Department of Cardiology, Affiliated Chinese Medicine Hospital of Xinjiang Medical University, Urumqi, Xinjiang, People’s Republic of China.

**Keywords:** Myocardial Infarctation, Inflammation, Interleukin-18, Interleukin-6, RNA, Long Noncoding, C-Reactive Protein, MicroRNAs, GKLF protein

## Abstract

**Introduction:**

This study investigated the correlation between the levels of long noncoding ribonucleic acids (lncRNAs) AF131217.1 and coronary slow flow (CSF).

**Methods:**

A total of 22 patients in the high-sensitivity C-reactive protein (hsCRP) group diagnosed with CSF from January 2018 to December 2018 were enrolled in this study. Coronary flow velocity was determined using the thrombolysis in myocardial infarction frame count (TFC) method. Results: LncRNA AF131217.1 expression in the CSF model was activated. Mean TFC was positively correlated with lncRNA AF131217.1 levels and hsCRP levels. LncRNA AF131217.1 induced inflammation factor levels in the in vitro model. Micro ribonucleic acid (miR)-128-3p is a target spot of lncRNA AF131217.1 on the inflammation in vitro model via Kruppel-like factor (KLF) 4. MiR-128-3p reduced inflammation factor levels (tumor necrosis factor alpha, interleukin [IL]-6, IL-1β, and IL-18). **Conclusion**: Thus, lncRNA AF131217.1 promoted inflammation in the regulated CSF via KLF4 by miR-128-3p.

**Table:** 

Abbreviations, acronyms & symbols				
ACS	= Acute coronary syndrome		LncRNAs	= Long noncoding ribonucleic acids
C/EBPβ	= CCAAT/enhancer binding protein beta		LPS	= Lipopolysaccharide
cDNA	= Complementary deoxyribonucleic acid		MiR	= Micro ribonucleic acid
CSF	= Coronary slow flow		mRNA	= Messenger ribonucleic acid
ELISA	= Enzyme-linked immunosorbent assay		Mut	= Mutant
GAPDH	= Glyceraldehyde-3-phosphate dehydrogenase		NF-κB	= Nuclear factor kappa B
hsCRP	= High-sensitivity C-reactive protein		RNA	= Ribonucleic acid
HUVECs	= Human umbilical vein endothelial cells		TFC	= Thrombolysis in myocardial infarction frame count
IL	= Interleukin		TNF-α	= Tumor necrosis factor alpha
KLF	= Kruppel-like factors		WT	= Wild type

## INTRODUCTION

The coronary slow flow (CSF) phenomenon is defined as the presence of delayed perfusion of peripheral coronary artery identified by coronary arteriography after excluding coronary artery disease, cardiomyopathy, valvular disease, congenital heart disease, connective tissue disease, etc. Although coronary angiography in patients with slow blood flow shows no stenosis of coronary artery, recurrent cardiovascular events still occur, generally clinically manifested by angina, arrhythmia, or acute coronary syndrome (ACS), which may be associated with CSF and usually requires emergency admission^[1^,^2]^. The exact pathological mechanism of CSF is still unclear. At present, there is no standardized therapeutic measures for CSF in clinical practice, with inconsistent reported efficacy of drug treatment^[2]^. Studies have shown that CSF is strongly associated with cardiovascular adverse events, such as arrhythmia, ACS, and sudden cardiac death. In addition, the levels of high-sensitivity C-reactive protein (hsCRP), vascular cell adhesion molecules, and intercellular adhesion molecules are significantly increased in CSF patients than in healthy individuals, which is also positively correlated with thrombolysis in myocardial infarction frame count (TFC), indicating endothelial activation and inflammatory response in CSF patients^[2^,^3]^.

Kruppel-like factors (KLF) are a type of transcription factors with zinc-finger structure, which is characterized by the three C2H2 zinc-finger structures at the carboxyl terminal^[4]^. KLF family members are widely involved in the regulation of multiple life activities, including cell proliferation, apoptosis, differentiation, and embryonic development^[5]^. The abnormal function of KLF family members is strongly associated with metabolic diseases, cardiovascular diseases, and cancer. KLF4, originally isolated from the gastrointestinal tract, is one of the transcriptional regulators required for the early differentiation of adipocytes^[6]^. It activates the expression of the CCAAT/enhancer binding protein beta (C/EBPβ) gene by binding itself to the promoter of C/EBPβ, thereby activating the downstream cascade of fat cell differentiation to promote this differentiation^[6^,^7]^. In recent years, KLF4 has become a research hotspot in relevant fields due to its regulatory roles on chronic inflammatory responses of various cells^[4]^.

Long noncoding ribonucleic acids (lncRNAs) are a type of noncoding RNA with over 200 bp in length^[8]^. To date, hundreds of thousands of eukaryotic lncRNAs have been found, most of which are lowly conservative and involved in the pathogenesis and progression of a series of diseases, including tumors, nervous system disorders, metabolic diseases, reproductive development, and cardiovascular diseases^[9]^. Long intergenic noncoding RNA-p21, a p53-induced lncRNA, is able to inhibit cell proliferation and promote apoptosis during atherosclerosis^[9^,^10]^. This study investigated the correlation between the levels of lncRNA AF131217.1 and CSF.

## METHODS

### Study Subjects and CSF Diagnosis

A total of 22 patients in the hsCRP group diagnosed with CSF at the Affiliated Chinese Medicine Hospital of Xinjiang Medical University, from January 2018 to December 2018, were enrolled in this study. All patients had stenosis of lumen diameter < 40% (no significant vascular lesion). The control group was 24 patients who did not meet the abovementioned criteria. All procedures performed in studies involving human participants were in accordance with the ethical standards of the institutional and/or National Research Committee and with the 1964 Helsinki Declaration and its later amendments or comparable ethical standards. This study was approved by the Ethics Committee of the Affiliated Chinese Medicine Hospital of Xinjiang Medical University. Written informed consent was obtained from all individual participants included in the study. TFC was used to quantitatively measure coronary blood flow using a cineangiography. CSF was diagnosed if TFC > 27 in at least one coronary artery. Three cardiologists who were blinded to the clinical findings independently assessed the TFC.

### LncRNA and micro RNA Analysis

Total RNA was purified with the RNeasy Kit (Qiagen). Complementary deoxyribonucleic acid (cDNA) and complementary RNA were generated and hybridized to HumanHT-12 v4 BeadChips (Illumina). Cubic spline-normalized (without background normalization) data were analyzed by the NIA Array Analysis tool (http://lgsun.grc.nia.nih.gov/ANOVA).

### Luciferase Reporter Assay

Micro RNA (miR)-128-3p was inserted into the luciferase gene psiCHECK2 vector, named as KLF4-WT (the wild type) or KLF4-Mut (mutant) 3'-UTR. Next, miR-128-3p was inserted into the luciferase gene psiCHECK2 vector, named as lncRNA AF131217.1-WT or lncRNA AF131217.1-Mut 3'-UTR. After transduction, at 48 hours, cell was transduced using Lipofectamine® 2000 Reagent (Thermo Fisher Scientific, Inc.). The luciferase assay was performed using Luciferase Reporter Gene Detection Kit (Sigma-Aldrich Co., St. Louis, Missouri, United States of America).

### Real-Time Reverse Transcription Polymerase Chain Reaction

Total RNA was isolated with the RNeasy Micro Kit (Qiagen) and cDNA was synthesized with the Maxima First Strand cDNA Synthesis Kit. Quantitative real-time polymerase chain reaction was performed using SYBR Green Master Mix. Relative fold changes were calculated using the 2^-△△Ct^ method.

### Cell Culture and Treatment

Human umbilical vein endothelial cells (HUVECs) were cultured in Roswell Park Memorial Institute 1640 medium (Thermo Fisher Scientific, Inc.) supplemented with 10% fetal bovine serum (Thermo Fisher Scientific, Inc.) at 37°C with 5% CO_2_. HUVECs (1×10^5^/well) were transiently transfected with 50 nM of lncRNA AF131217.1, silencing lncRNA AF131217.1, miR-128-3p, and negative control using Lipofectamine® 2000 reagent (Invitrogen; Thermo Fisher Scientific, Inc.). After 48 hours of transfection, HUVECs were treated with 100 µg/ml oxidized low-density lipoprotein for 24 hours.

### Western Blots

Cells were lysed with radioimmunoprecipitation buffer, and protease and phosphatase inhibitors (Sigma-Aldrich) were added. Protein concentration was measured using a bicinchoninic acid protein assay kit (Beyotime, Jiangsu, China). Equal amounts of proteins were separated by 10% sodium dodecyl sulphate-polyacrylamide gel electrophoresis and transferred to polyvinylidene ﬂuoride membranes. After blocking with no-fat skim milk, the membranes were incubated with primary antibodies (KLF4, RhoF, nuclear factor kappa B [NF-κB], and glyceraldehyde-3-phosphate dehydrogenase) diluted in Tris Buffered Saline with Tween 20 overnight at 4°C and then with the horseradish peroxidase-conjugated secondary antibody for two hours at room temperature. Protein bland was detected by electrochemiluminescence chromogenic substrate kits and quantified by Image Lab 3.0 (Bio-Rad Laboratories, Inc.).

### Enzyme-linked Immunosorbent Assay (ELISA) Kit Analysis

Serum samples were collected at 1000 g for 10 min and used to measure tumor necrosis factor alpha (TNF-α), interleukin (IL)-6, IL-1β, and IL-18 levels. Cell samples were also collected at 1000 g for 10 min and used to measure TNF-α, IL-6, IL-1β, and IL-18 levels. TNF-α, IL-6, IL-1β, and IL-18 ELISA kits were purchased from Nanjing Jiancheng Biological Engineering Research Institute Co. LTD (Nanjing, China).

### Statistical Analysis

All data are expressed as the mean ± standard error of mean. P<0.05 was considered statistically significant. Statistical significance between groups of data was calculated by Student’s t-test or one-way analysis of variance and Tukey's post-test.

## RESULTS

### LncRNA AF131217.1 Expression in the CSF Model

**Fig. 1:**
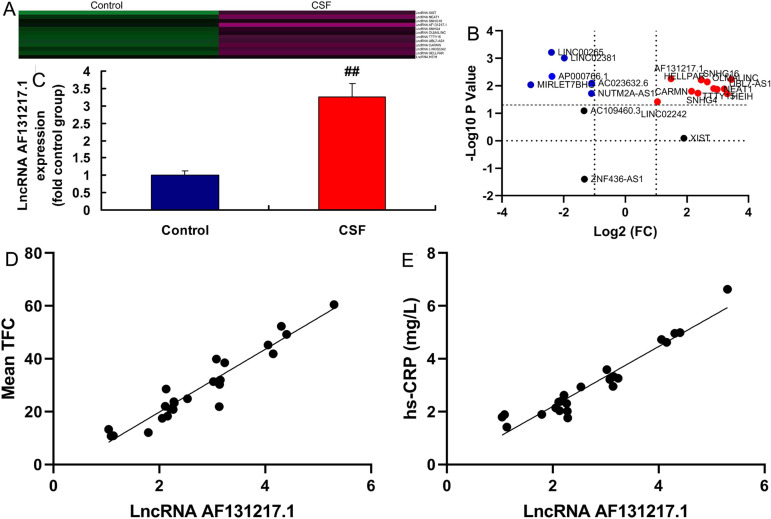
Long noncoding ribonucleic acid (lncRNA) AF131217.1 expression in the coronary slow flow (CSF) model. Heat map and volcano figure of gene chip (A and B), quantitative real-time polymerase chain reaction of lncRNA AF131217.1 expression (C) in the CSF model. The mean thrombolysis in myocardial infarction frame count (TFC) was positively correlated with lncRNA AF131217.1 levels (D) and high-sensitivity C-reactive protein (hsCRP) levels (E). Control, normal volunteer group; hsCRP, hsCRP patients group. ^##^P<0.01 compared with normal volunteer group.

Initially, we sought to validate lncRNA AF131217.1 expression in the CSF model. We found using gene chip that six genes were downregulated and 11 genes were upregulated in the CSF model (Figures 1A and 1B). LncRNA AF131217.1 expression in the CSF model was activated, compared with the control group (Figure 1C). Mean TFC was positively correlated with lncRNA AF131217.1 levels (Figure 1D) and hsCRP levels (Figure 1E).

### LncRNA AF131217.1 Regulated Inflammation in the In Vitro Model

**Fig. 2:**
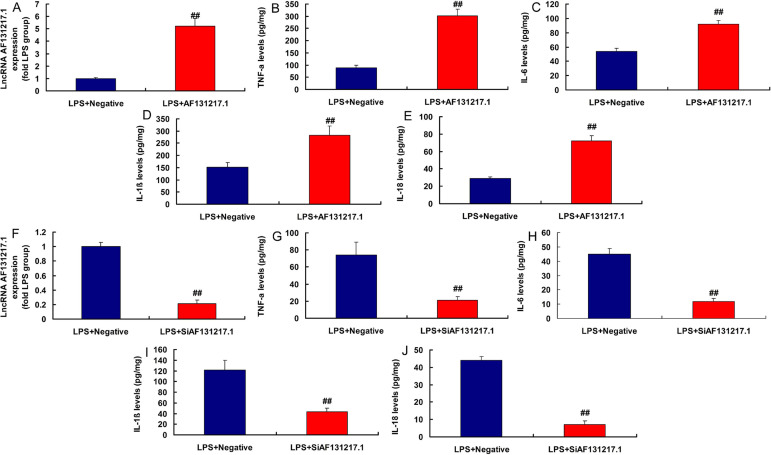
Long noncoding ribonucleic acid (lncRNA) AF131217.1 regulated inflammation in the in vitro model. LncRNA AF131217 expression (A), tumor necrosis factor alpha (TNF-α), interleukin (IL)-6, IL-1β, and IL-18 (B, C, D, and E) in overexpression of lncRNA AF131217 group; lncRNA AF131217 expression (F), TNF-α, IL-6, IL-1β, and IL-18 (G, H, I, and J) in downregulation of lncRNA AF131217 group. LPS+Negative, LPS-induced in vitro model by negative mimics group; LPS+AF131217.1, LPS-induced in vitro model by AF131217.1 group. ^##^P<0.01 compared with LPS+Negative group. LPS=lipopolysaccharide.

Moreover, in the lipopolysaccharide (LPS)-induced in vitro model, lncRNA AF131217.1 plasmid increased lncRNA AF131217.1 expression and induced inflammation factor levels (TNFα, IL-6, IL-1β, and IL-18) (Figures 2A to 2E). Then, in LPS-induced in vitro model, silencing lncRNA AF131217.1 decreased lncRNA AF131217.1 expression and reduced inflammation factor levels (TNFα, IL-6, IL-1β, and IL-18) (Figure 2F to 2J). Thus, lncRNA AF131217.1 expression in the CSF model was upregulated and started an inflammatory reaction.

### MiR-128-3p is a Target Spot of lncRNA AF131217.1 on the Inflammation In Vitro Model

**Fig. 3:**
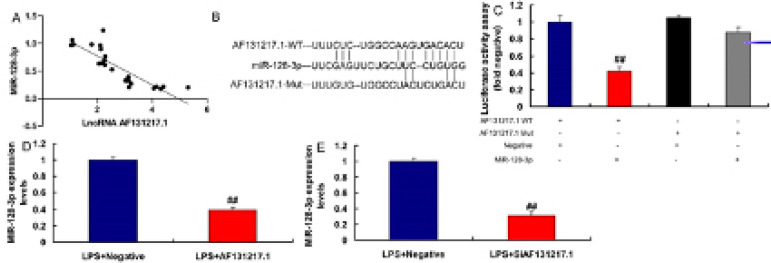
Micro ribonucleic acid (miR)-128-3p is a target spot of long noncoding ribonucleic acid (lncRNA) AF131217.1 on the inflammation in vitro model. MiR-128-3p expression had a negative correlation with lncRNA AF131217.1 levels in the coronary slow flow model (A). The luciferase reporter plasmid containing wild type (WT) or mutant (Mut) AF131217.1 was cotransfected with miR-128-3p (B and C), overexpression of lncRNA AF131217.1 suppressed miR-128-3p expression (D), downregulation of lncRNA AF131217.1 induced miR-128-3p expression in lipopolysaccharide (LPS)-induced in vitro model (E). LPS+Negative, LPS-induced in vitro model by negative mimics group; LPS+AF131217, LPSinduced in vitro model by AF131217.1 group. ^##^P<0.01 compared with LPS+Negative group.

In our search to define the possible target spot of lncRNA AF131217.1 on inflammation of CSF, miR-128-3p expression had a negative correlation with lncRNA AF131217.1 levels in the CSF model (Figure 3A). The relative luciferase activity in AF131217.1-WT and miR-128-3p groups was lower than that of AF131217.1-Mut and miR-NC cotransfection groups (Figures 3B and 3C). Nonetheless, overexpression of lncRNA AF131217.1 suppressed miR-128-3p expression and downregulation of lncRNA AF131217.1 induced miR-128-3p expression in LPS-induced in vitro model (Figures 3D and 3E).

### MiR-128-3p and KLF4 Expressions in the CSF Model

**Fig. 4:**
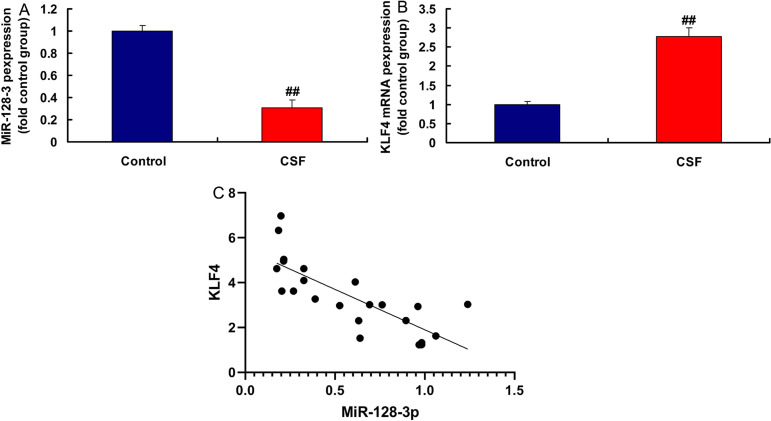
Micro ribonucleic acid (MiR)-128-3p and Kruppel-like factor (KLF) 4 expressions in the coronary slow flow (CSF) model (A and B), miR- 128-3p expression had a negative correlation with KLF4 levels in the rat model (C). Control, normal volunteer group; CSF, CSF patients group. ^##^P<0.01 compared with normal volunteer group. mRNA=messenger ribonucleic acid

**Fig. 5:**
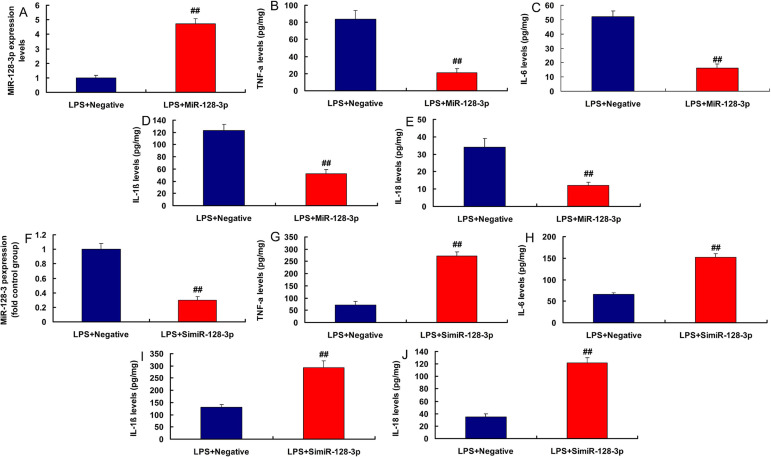
Micro ribonucleic acid (MiR)-128-3p regulated inflammation in the in vitro model. MiR-128-3p expression (A), tumor necrosis factor alpha (TNF-α), interleukin (IL)-6, IL-1β, and IL-18 (B, C, D, and E) in overexpression of miR-128-3p group; miR-128-3p expression (F), TNF-α, IL-6, IL-1β, and IL-18 (G, H, I, and J) in downregulation of miR-128-3p group. LPS+Negative, LPS-induced in vitro model by negative mimics group; LPS+miR-128-3p, LPS-induced in vitro model by miR-128-3p group. ^##^P<0.01 compared with LPS+Negative group. LPS=lipopolysaccharide

In the rat model of CSF, miR-128-3p expression was downregulated and KLF4 expression was upregulated, compared with the control group (Figures 4A and 4B). MiR-128-3p expression had a negative correlation with KLF4 levels in the rat model (Figure 4C). Moreover, in LPS-induced in vitro model, miR-128-3p plasmid increased miR-128-3p expression and reduced inflammation factor levels (TNFα, IL-6, IL-1β, and IL-18) (Figures 5A to 5E). Then, in LPS-induced in vitro model, simiR-128-3p AF131217.1 decreased miR-128-3p expression and promoted inflammation factor levels (TNFα, IL-6, IL-1β, and IL-18) (Figures 5F to 5J). Thus, miR-128-3p expression in the CSF model was downregulated and revealed anti-inflammatory effect.

### KLF4 is a Target Spot of miR-128-3p on the Inflammation In Vitro Model

**Fig. 6:**
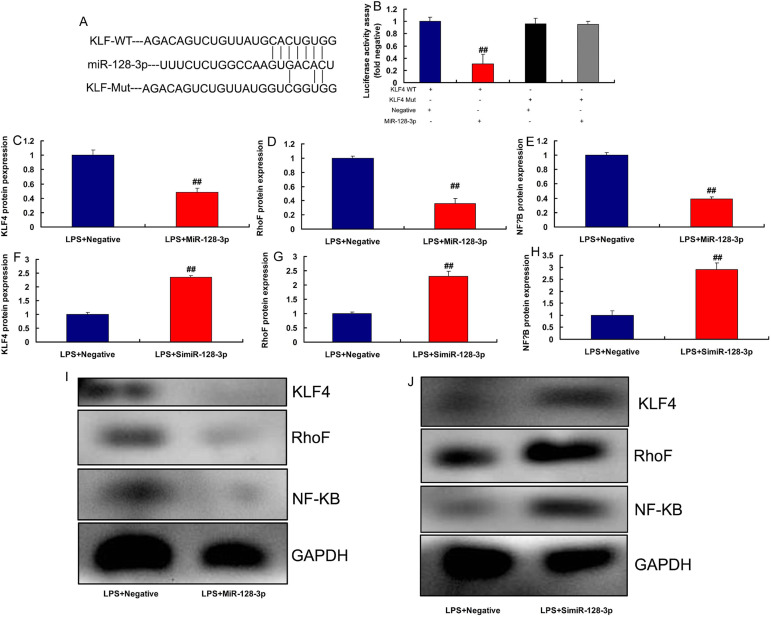
Kruppel-like factor (KLF) 4 is a target spot of micro ribonucleic acid (MiR)-128-3p on the inflammation in vitro model. The luciferase reporter plasmid containing wild type (WT) or mutant (Mut) KLF4 was cotransfected with miR-128-3p (A and B), KLF4, RhoF, and nuclear factor kappa B (NF-κB) protein expressions (C, D, E, and I) by overexpression of miR-128-3p; KLF4, RhoF, and NF-κB protein expressions (F, G, H, and J) by downregulation of miR-128-3p. LPS+Negative, LPS-induced in vitro model by negative mimics group; LPS+miR-128-3p, LPS-induced in vitro model by miR-128-3p group; LPS+simiR-128-3p, LPS-induced in vitro model by simiR-128-3p group. ^##^P<0.01 compared with LPS+Negative group. GAPDH=glyceraldehyde-3-phosphate dehydrogenase; LPS=lipopolysaccharide

The study defined the possible target spot of miR-128-3p on inflammation of CSF, the relative luciferase activity in KLF4-WT and miR-128-3p groups was lower than that of KLF4-Mut and miR-NC cotransfection groups (Figures 6A and 6B). Nonetheless, overexpression of miR-128-3p suppressed KLF4, RhoF, and NF-κB protein expressions (Figures 6C to 6E, 6I). Downregulation of miR-128-3p induced KLF4, RhoF, and NF-κB protein expressions in LPS-induced in vitro model (Figures 6F to 6H, 6J).

LncRNA AF131217.1 Regulated Inflammation in the In Vitro Model via KLF4 by miR-128-3p

**Fig. 7:**
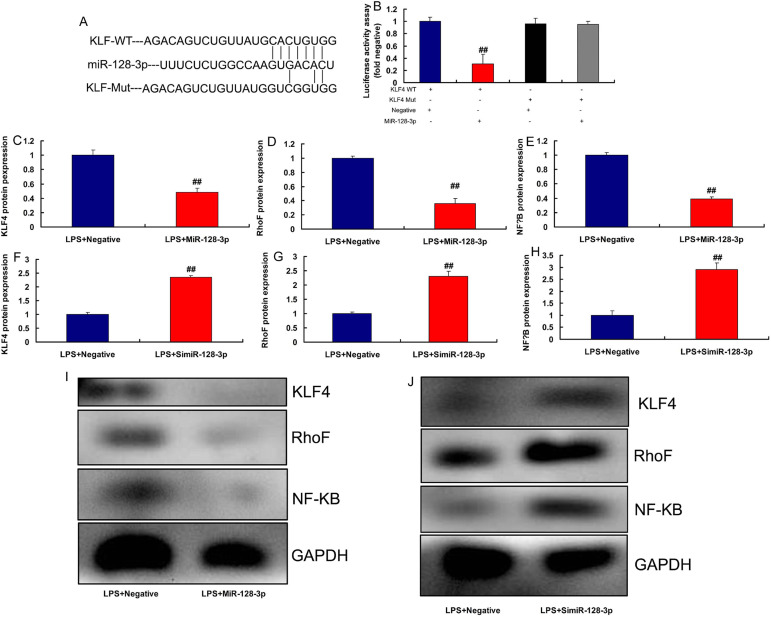
Long noncoding ribonucleic acid AF131217.1 regulated inflammation in the in vitro model via Kruppel-like factors (KLF) 4 by micro ribonucleic acid (miR)-128-3p. MiR-128-3p expression (A), tumor necrosis factor alpha (TNF-α), interleukin (IL)-6, IL-1β, IL-18, KLF4, RhoF, and nuclear factor kappa B (NF-κB) protein expressions (B, C, D, E, F, G, H, and I). LPS+Negative, LPS-induced in vitro model by negative mimics group; LPS+AF131217, LPS-induced in vitro model by AF131217.1 group; LPS+AF131217+miR-128-3p, LPS-induced in vitro model by AF131217.1 and miR-128-3p group. ## P<0.01 compared with LPS+Negative group; ** P<0.01 compared with LPS+ AF131217.1 group. GAPDH=glyceraldehyde- 3-phosphate dehydrogenase; LPS=lipopolysaccharide

In an attempt to delineate whether miR-128-3p was required for lncRNA AF131217.1 induction of KLF4 signaling in CSF, we found that miR-128-3p overexpression induced miR-128-3p expression and reduced inflammation factor levels (TNFα, IL-6, IL-1β, and IL-18) in the in vitro model of lncRNA AF131217.1 (Figure 7A-7E). Meanwhile, miR-128-3p overexpression suppressed KLF4, RhoF, and NF-κB protein expressions in the in vitro model of lncRNA AF131217.1 (Figure 7F-7I).

## DISCUSSION

CSF is defined as the presence of delayed perfusion of peripheral coronary artery but without obvious lesions in the coronary arteries diagnosed by coronary arteriography after excluding coronary spasm, coronary artery dilatation, coronary angioplasty, cardiomyopathy, heart valvular disease, autoimmune diseases, tumors, and other important organs or systemic diseases. More and more attention are being paid to the research of CSF, however, the etiology and pathogenesis of CSF remain unclear^11^-^13]^. We showed that lncRNA AF131217.1 expression in the CSF model was activated. Mean TFC was positively correlated with lncRNA AF131217.1 levels and hsCRP levels. These characteristics suggest that lncRNA AF131217.1 participated in the pathophysiological process of CSF.

Lu et al.^[14]^ showed that shear-sensitive lncRNA AF131217.1 reduced inflammation in HUVECs via regulation of KLF4.

Cardiovascular disease is a common disease that threatens the health of human beings, especially the elderly^[15]^. The annual number of people dying from cardiovascular disease ranks the first globally^[15]^. LncRNAs are a type of noncoding RNAs with over 200 nt in length, which are also the most abundantly expressed RNA^[16^,^17]^. Accumulative studies have revealed that lncRNAs play an important regulatory role in the pathogenesis and development of cardiovascular diseases^[16]^. Interestingly, lncRNA AF131217.1 induced inflammation factor levels and played an important role in the inflammation of CSF.

Wang et al.^[18]^ showed that miR-128-3p accelerates cardiovascular calcification in type 2 diabetes mellitus rats. Studies have shown that CSF is closely associated with cardiovascular adverse events, including arrhythmia, ACS, and sudden cardiac death^[19^,^20]^. When coronary angiography is performed on patients with suspected cardiovascular disease, the detection rate of CSF is approximately 1%^[19]^. Recent studies have demonstrated that inflammatory response plays an important role in the pathogenesis of CSF^[19^,^21]^. Our study showed that miR-128-3p is a target spot of lncRNA AF131217.1 on the inflammation in vitro model.

KLF4 has been reported to physically bind to the active subunit P65 of transcription factor NF-κB in vascular endothelial cells to prevent the nuclear translocation of P65, thereby exerting its anti-inflammatory role by inhibiting the transcriptional activation on downstream inflammatory factors of P65^[22]^. In endothelial cells and immune cells, P300 can bind with the active subunit P65 of NF-κB to induce acetylation of P65, thereby promoting the transcriptional activation on downstream inflammatory factors by P65^[23]^. Overexpressed KLF4 can competitively bind to P300, inhibiting the binding of P300 with P65 to attenuate its transcriptional activity, subsequently exerting an anti-inflammatory effect^[23]^. As members of the KLF family, KLF2 and KLF15 can also competitively bind with P300^[24]^. It has been reported that KLF4 can bind to the active subunit P65 of NF-κB in vascular endothelial cells, to promote the binding of P65 to its downstream inflammatory factor vascular cell adhesion molecule 1, thereby playing a pro-inflammatory role^[25]^. Meanwhile, we found that KLF4 is a target spot of miR-128-3p on the inflammation in vitro model.

## CONCLUSION

In summary, lncRNA AF131217.1 expression in the CSF model was activated and promoted inflammation by the suppression of miR-128-3p via KLF4/RhoF/NF-κB signal pathway, and this may play an important role in the pathogenesis of CSF. An elevated plasma lncRNA AF131217.1 level may indicate the presence of CSF. Further studies are needed to establish clinical significance of increased plasma lncRNA AF131217.1 levels and to investigate the therapeutic efficacy of targeting miR-128-3p/KLF4/RhoF/NF-κB signal pathway.

**Table:** 

Authors' roles & responsibilities	
ZG	Substantial contributions to the acquisition of data for the work; revising the work for important intellectual content; final approval of the version to be published
LZ	Substantial contributions to the acquisition of data for the work; final approval of the version to be published
YY	Substantial contributions to the acquisition of data for the work; final approval of the version to be published
XZ	Substantial contributions to the analysis of data for the work; final approval of the version to be published
ZC	Drafting the work and revising it; final approval of the version to be published
QW	Revising the work; final approval of the version to be published
HJ	Substantial contributions to the conception or design of the work; agreement to be accountable for all aspects of the work in ensuring that questions related to the integrity of any part of the work are appropriately investigated and resolved; final approval of the version to be published

## References

[1] Aydinli B, Demir A, Özmen H, Vezir Ö, Ünal U, Özdemir M (2018). Koroner Cerrahisinde Preoperatif HbA1c Degerleri Mortalite Icin
Prediktor Olabilir mi. Turk J Anaesthesiol Reanim.

[2] Almogati JG, Ahmed EO (2019). Glycated Hemoglobin as a Predictor of the Length of Hospital Stay
in Patients Following Coronary Bypass Graft Surgery in the Saudi
Population. Braz J Cardiovasc Surg.

[3] Ouattara A, Lecomte P, Le Manach Y, Landi M, Jacqueminet S, Platonov I (2005). Poor intraoperative blood glucose control is associated with a
worsened hospital outcome after cardiac surgery in diabetic
patients. Anesthesiology.

[4] Lazar HL, Chipkin SR, Fitzgerald CA, Bao Y, Cabral H, Apstein CS. (2004). Tight glycemic control in diabetic coronary artery bypass graft
patients improves perioperative outcomes and decreases recurrent ischemic
events. Circulation.

[5] Peters AL, Davidson MB, Schriger DL, Hasselblad V (1996). A clinical approach for the diagnosis of diabetes mellitus: an
analysis using glycosylated hemoglobin levels. Meta-Research Group on the
Diagnosis of Diabetes Using Glycated Hemoglobin Levels. JAMA.

[6] Tennyson C, Lee R, Attia R. (2013). Is there a role for HbA1c in predicting mortality and morbidity
out comes after coronary artery bypass graft surgery?. Interact Cardiovasc Thorac Surg.

[7] Faritous Z, Ardeshiri M, Yazdanian F, Jalali A, Totonchi Z, Azarfarin R (2014). Hyperglycemia or high hemoglobin A1C: Which one is more
associated with morbidity and mortality after coronary artery bypass graft
surgery?. Ann Thorac Cardiovasc Surg.

[8] Li Z, Amsterdam EA, Young JN (2015). Contemporary outcomes of coronary artery bypass grafting among
patients with insulin-treated and non-insulin-treated
diabetes. Ann Thorac Surg.

[9] Najafi M, Goodarzynejad H. (2012). Determinants of length of stay in surgical ward after coronary
bypass surgery: glycosylated hemoglobin as a predictor in all patients,
diabetic or non-diabetic. J Tehran Heart Cent.

[10] Medhi M, Marshall MC, Burke HB, Hasan R, Nayak D, Reed G (2001). HbA1c predicts length of stay in patients admitted for coronary
artery bypass surgery. HeartDis.

[11] Khan MR, Khan H, Wahab A, Chaudharya S, Munirb A (2019). Effect of glycemic control on mortality and infections in
patients undergoing coronary artery bypass grafting: a Genesee County
experience. Journal of community hospital internal medicine perspectives.

[12] Okusa MD, Davenport A (2014). Reading between the guidelines: the KDIGO practice guideline on
acute kidney injury in the individual patient. Kidney Int.

[13] Latham R, Lancaster AD, Covington JF, Pirolo JS, Thomas CS (2001). The association of diabetes and glucose control with surgical
site infections among cardiothoracic surgery patients. Infect Control Hosp Epidemiol.

[14] Furnary AP, Gao G, Grunkemeier GL (2003). Continuous insulin infusion reduces mortality in patients with
diabetes undergoing coronary artery bypass grafting. J Thorac Cardiovasc Surg.

[15] Knapik P, Ciesla D, Filipiak K, Knapik M, Zembala M (2011). Prevalence and clinical significance of elevated preoperative
glycosylated hemoglobin in diabetic patients scheduled for coronary artery
surgery. Eur J Cardiothorac Surg.

[16] Matsuura K, Imamaki M, Ishida A, Shimura H, Niitsuma Y, Miyazaki M (2009). Off-pump coronary artery bypass grafting for poorly controlled
diabetic patients. Ann Thorac Cardiovasc Surg.

[17] Kuhl J, Sartipy U, Eliasson B, Nyström T, Holzmann MJ (2016). Relationship between preoperative hemoglobin A1c levels and
long-term mortality after coronary artery bypass grafting in patients with
type 2 diabetes mellitus. Int J Cardiol.

[18] Halkos ME, Puskas JD, Lattouf OM, Kilgo P, Kerendi F, Song HK (2008). Elevated preoperative hemoglobin A1c levelis predictive of
adverse events after coronary artery bypass surgery. J Thorac Cardiovasc Surg September.

[19] Cohen O, Dankner R, Chetrit A, Luxenburg O, Langenauer C, Shinfeld A (2003). Multidisciplinary intervention for control of diabetes in
patients undergoing coronary artery bypass graft(CABG). Cardiovasc Surg.

[20] Beattie WS, Wijeysundera DN (2013). Perioperative cardiac biomarkers: the utility and
timing. Curr Opin Crit Care.

[21] Akram R. Allama, Magdy A. Sorourb, Mohamed M. Aghaa. (2018). Renal dysfunction after coronary artery bypass
surgery. Research and Opinion in Anesthesia&Intensive Care.

[22] Regner KR, Connolly HM, Schaff HV, Albright RC (2005). Acute renal failure after cardiac surgery for carcinoid heart
disease: incidence, risk factors, and prognosis. Am J KidneyDis.

[23] Sevük U, Bilgiç A, Yaylak B, Ay N, Baysal E, Altindag R, Alp V, Beyazit Ü, Akkaya S, Erkul A (2016). Relationship Between Elevated HbA1c and Deep Sternal Wound
Infection in Patients Undergoing Cardiac Surgery. Kosuyolu Heart Journal.

[24] Finger B, Brase J, He J, Gibson WJ, Wirtz K, Flynn BC (2017). Elevated hemoglobin A1c is associated with lower socioeconomic
position and increased postoperative infections and longer hospital stay
after cardiac surgical procedures. Ann Thorac Surg.

[25] Hadjinikolaou L, Klimatsidas M, Maria Iacona G, Spyt T, Samani NJ (2010). Short and medium term survival following coronary artery bypass
surgery in British Indo-Asian and White Caucasian individuals: impact of
diabetes melli-tus. Interact Cardiovasc Thorac Surg.

